# Hyperuricemia after orthotopic liver transplantation: divergent associations with progression of renal disease, incident end-stage renal disease, and mortality

**DOI:** 10.1186/s12882-017-0518-5

**Published:** 2017-03-27

**Authors:** Joseph C. Longenecker, Sana Waheed, Ghassan Bandak, Christine A. Murakami, Blaithin A. McMahon, Allan C. Gelber, Mohamed G. Atta

**Affiliations:** 1 Department of Community Medicine and Behavioural Sciences, Kuwait University Faculty of Medicine, Kuwait City, Kuwait; 2 Department of Medicine, Johns Hopkins University School of Medicine, Baltimore, MD USA; 3 Department of Medicine, University of Wisconsin School of Medicine, Madison, WI USA; 4 Department of Medicine, Mayo Clinic, Rochester, MN USA; 5 Lakelands Nephrology, PA, Greenwood, SC USA

**Keywords:** Uric acid, Liver transplantation, eGFR, Clinical epidemiology

## Abstract

**Background:**

Although hyperuricemia is common after orthotopic liver transplantation (OLT), its relationship to mortality, progressive kidney disease, or the development of end stage renal disease (ESRD) is not well-described.

**Methods:**

Data from 304 patients undergoing OLT between 1996 and 2010 were used to assess the association of mean serum uric acid (UA) level in the 3-months post-OLT with mortality, doubling of creatinine, and ESRD incidence. Post-OLT survival to event outcomes according to UA level and eGFR was assessed using the Kaplan Meier method and multivariate Cox proportional hazards models.

**Results:**

Mean UA level among the 204 patients with an eGFR level ≥60 ml/min/1.73 m^2^ was 6.4 mg/dl compared to 7.9 mg/dl among the 100 patients with eGFR <60 (*p* < 0.0001). During a median of 4.6 years of follow-up, mortality rate, doubling of creatinine, and ESRD incidence were 48.9, 278.2, and 20.7 per 1000 person-years, respectively. In the first 5 years of follow-up, elevated UA was associated with mortality (Hazard Ratio, HR = 1.7; *p* = 0.045). However, among those with eGFR ≥ 60, UA level did not predict mortality (HR = 1.0; *p* = 0.95), and among those with eGFR < 60, elevated UA was a strong predictor of mortality (HR = 3.7[1.1, 12.0]; *p* = 0.03). UA was not associated with ESRD, but was associated with doubling of creatinine among diabetics (HR = 2.2[1.1, 4.3]; *p* = 0.025).

**Conclusion:**

In this post-OLT cohort, hyperuricemia independently predicted mortality, particularly among patients with eGFR < 60, and predicted doubling of creatinine among diabetics.

**Electronic supplementary material:**

The online version of this article (doi:10.1186/s12882-017-0518-5) contains supplementary material, which is available to authorized users.

## Background

Hyperuricemia is a common occurrence after organ transplantation, [1] with a high prevalence after cardiac/lung transplantation (70–80%), [2] renal transplantation (30–40%), [1] and liver transplantation (14 to 47%) [3]. Uric acid (UA) is handled in the kidney by membrane transport proteins in the proximal tubules [4]. Therefore, hyperuricemia often accompanies declining renal function, both of which are commonly seen in the post-OLT population [5].

Several studies have reported an association between hyperuricemia and cardiovascular mortality in the general population [6, 7], and in patients with chronic kidney disease (CKD) [8]. General population studies have also reported that hyperuricemia is prospectively associated with worsening renal disease and progression to ESRD [9]. However, a recent study of patients with moderate to severe CKD found that hyperuricemia is not prospectively associated with decline in renal function [10].

In the liver transplant population, some studies have suggested that hyperuricemia in the post-transplant period may be an independent predictor of renal disease after orthotropic liver transplantation (OLT) [11]. Furthermore, the treatment of hyperuricemia in liver transplant recipients is associated with an improvement in kidney function, suggesting that uric acid may be a cause of renal disease in this population [3]. Small studies in non-transplant populations have also found that allopurinol lowers blood pressure, reduces left ventricular hypertrophy and preserves renal function [12, 13].

Although hyperuricemia has been associated with increased mortality in the post-renal transplantation period, [14, 15] little is known about the impact of hyperuricemia on mortality and progression of renal disease in the post-liver transplantation period, particularly in those with concomitant CKD. The objective of this cohort study is to assess hyperuricemia and its prospective association with mortality, doubling of creatinine, and progression to ESRD among orthotopic liver transplant recipients.

## Methods

This non-concurrent cohort study included 304 patients who underwent first OLT between January, 1996, and February, 2009, at a single U.S. tertiary hospital with an active liver transplantation program. Exclusion criteria included age <18 years, transplantation of multiple organs, and status 1A liver failure. Of 481 OLT patients eligible for inclusion in the study, 304 patients had serum uric acid measurements obtained during the 90-day period after OLT. Four patients who had been enrolled in the Medicare dialysis program before OLT were excluded from the analysis of ESRD incidence and doubling of creatinine (*n* = 300). The study protocol was approved by the Johns Hopkins School of Medicine Institutional Review Board.

Clinical data were obtained by medical record review, including demographic factors, liver disease etiology, and co-morbid conditions (all defined by documentation in the medical record). Uric acid and serum creatinine levels assayed by the John Hopkins Hospital Laboratory using standard clinical laboratory techniques were retrieved from the laboratory database. The Model for End-Stage Liver Disease (MELD) score at OLT was calculated in accordance with the United Network for Organ Sharing (UNOS) formula [16]. To calculate the MELD score, serum bilirubin, creatinine, and International Normalized Ratio (INR) values less than 1.0 were set to 1.0 to preclude negative values, and serum creatinine upper-limit values were set at 4.0 if the patient required renal replacement therapy prior to transplantation. Renal function was assessed by estimating glomerular filtration rate (eGFR) using the Chronic Kidney Disease Epidemiology Collaboration (CKD-EPI) formula [17]. Categories of eGFR were created according to standard classifications [18]. Information regarding the primary calcineurin inhibitor (CNI) used after transplantation was obtained in 2015 from a review of electronic discharge summaries for the OLT admission. However, discharge summaries for admissions before 2003 were not available. Therefore, for those admissions, the type of CNI in use during 2003/4 was recorded, if available.

Three primary study outcomes included total mortality, doubling of creatinine, and ESRD incidence from time of OLT with follow-up through April 30, 2010. The primary exposure variable was the mean of all UA levels obtained during the 3 months after OLT. For participants with death (*n* = 5) or an ESRD event (*n* = 7) that occurred during the 3 months post-OLT, the mean uric acid level between OLT and the event was used. A total of 3425 uric acid levels obtained during the 3 months after OLT were available for analysis. A median of 4 [IQR: 2–9; Mean: 11] uric acid measurements were available per patient. Doubling of creatinine was defined as attaining a creatinine level more than twice the nadir creatinine level within the first 90 days post-OLT. For women with a nadir creatinine level <0.5 mg/dl and men <0.6 mg/dl, the nadir creatinine level was set to 0.5 mg/dl and 0.6 mg/dl, respectively.

Vital status was assessed using data from the National Death Index, electronic medical record, and UNOS registry. Time to death was assessed as time from OLT to death. Data on occurrence of end stage renal disease, as measured by enrollment in the Medicare dialysis program, were obtained by linkage of the clinical dataset to the United States Renal Data System (USRDS).

### Statistical analysis

Data were analyzed using Stata/SE 12 (StataCorp, College Station, TX). The Student *t*-test and Analysis of Variance (ANOVA) were used to assess unpaired associations between continuous and categorical variables, where appropriate. A two-sided p-value of less than 0.05 was considered significant. Survival analysis was used to assess the association of the mean uric acid level during the 3 months post-OLT with time to death and time to ESRD incidence. Incidence rates, 95% confidence intervals, and significance levels were generated using the Stata *stset, stptime* and *stir* commands. The Kaplan-Meier product limit estimator function was used to assess survival according to uric acid level, dichotomized at 6.5 mg/dl. We chose the same uric acid cut-point for male and female patients since the median uric acid level essentially equalized in this population and did not differ significantly according to gender in the 3 months after OLT. The log-rank and Breslow tests were used to assess statistical significance between survival curves. Cox proportional hazards models were constructed (with time from OLT to death, doubling of creatinine, or ESRD as dependent variables) to estimate unadjusted and adjusted hazard ratios (HR) of all-cause mortality and ESRD following transplantation and related 95% confidence intervals (95% CI). For the mortality analyses, multivariate Cox regression models adjusted for age, sex, and eGFR level after OLT, the latter as a time-varying covariate for 12 months after OLT (diabetes was not associated with total mortality, and therefore was not included in the mortality models). For the doubling of creatinine and ESRD incidence analyses, the same approach was used, except that diabetes was also included as a clinically-important covariate. A priori analyses stratified by baseline pre-OLT eGFR level (mean eGFR over the 14 days prior to OLT, dichotomized at 60 mg/min/1.73 m^2^) were performed for the mortality, doubling of creatinine, and ESRD outcomes, and an interaction term (uric acid X eGFR level) was entered into the models to test the statistical significance of interaction. Additionally, for the doubling of creatinine and ESRD analyses, a priori stratification by diabetes with a corresponding test for interaction (uric acid X diabetes) was conducted. All models adhered to the proportionality assumption, as assessed by Schoenfeld residuals, a global test of proportionality, and log-log survival plots.

## Results

The mean age was 51.5 years, and 65.5% of the transplant recipients were male (Table 1). Approximately 60% of OLT patients had a MELD score below 20; 68% had an eGFR ≥60 ml/min/1.73 m^2^, and 28% were diabetic. To assess the representativeness of the 304 patients included in this study, comparison was made with the eligible patients (*n* = 177) who similarly underwent OLT during the same calendar period but did not have any uric acid levels measured during the 3-month period after OLT. The study group was comparable to the group without uric acid values with respect to mean age (52 vs. 52, respectively), male sex (66% vs. 68%), white race (71% vs. 74%), prevalent diabetes (28% vs. 27%), hypertension (19% vs. 25%), and median eGFR during the year after OLT (70 vs. 67 ml/min/1.73 m^2^). These findings suggest the 304 patients included in the study are reasonably representative of the 481 eligible OLT patients.

**Table 1:** Demographic and clinical characteristics at time of liver transplantation, Johns Hopkins Hospital, 1996–2009

Characteristic	Frequency	
	n	(%)
Age (years)		
≤ 45	58	(19.1)
46–54	134	(44.1)
≥ 55	112	(36.8)
Mean age (±s.d.)	51.5	(±9.4)
Gender (% male)	199	(65.5)
Race		
White	216	(71.1)
Black	63	(20.7)
Other	25	(8.2)
Cause of liver failure		
HCV	142	(46.9)
HBV	14	(4.6)
Alcohol	30	(9.9)
Other	117	(38.6)
MELD Era		
Pre-MELD era (before 2/2002)	151	(49.7)
MELD era (after 2/2002)	153	(50.3)
MELD score category		
< 10	11	(3.7)
10–19	166	(55.3)
20–29	85	(28.3)
30–39	32	(10.7)
≥ 40	6	(2.0)
Mean MELD Score (±s.d.)	20.3	(±7.6)
eGFR Category at Tx		
≥ 90	138	(46.0)
60–89	66	(22.0)
30–59	65	(21.7)
< 30	31	(10.3)
Mean Pre-Tx eGFR-Epi (±s.d.)	79.4	(±34.5)
Hepatorenal syndrome	20	(6.9)
Hemodialyzed before OLT Transplantation	8	(2.8)
Hemodialyzed after OLT	13	(4.5)
Diabetes	79	(27.5)
Hypertension	55	(19.4)
Hepatitis C Virus	139	(48.1)
Hepatocellular Carcinoma	22	(7.2)
Mycophenolate Mofetil	145	(53.9)
Steroids	259	(92.5)
Allopurinol	1	(0.6)
Calcineurin Inhibitor		
Tacrolimus	204	(67.1)
Cyclosporine	20	(6.6
None	8	(2.6)
Type not known	72	(23.7)

The overall mean (±s.d.) uric acid level was 6.9 (±2.3) mg/dl., and the median [interquartile range] was 6.6 [5.3, 8.4] mg/dl (Table 2). Uric acid was strongly associated with lower eGFR in the 3-month period after OLT. Although older age, white race, higher MELD score, hypertension, and presence of Hepatitis C were all associated with higher uric acid levels, these associations did not reach statistical significance. Other characteristics were not associated with uric acid level.

**Table 2:** Mean serum uric acid levels in the 3-month period after liver transplantation, according to patient characteristics

	Mean serum uric acid (mg/dl)		
Characteristic	n	Mean (±SD)	P
All	304	6.9 (±2.3)	
		[median, 6.6]	
Age (years)			0.08
≤ 50	119	6.6 (±2.3)	
> 50	185	7.1 (±2.3)	
Gender			0.28
Male	199	7.0 (±2.3)	
Female	105	6.7 (±2.3)	
Race			0.07
White	216	7.1 (±2.3)	
Black/Other	88	6.6 (±2.3)	
MELD era			0.27
Before 2/2002	151	6.8 (±2.2)	
After 2/2002	153	7.1 (±2.3)	
eGFR Category^a^ at Tx			<0.0001
≥ 90	138	6.0 (±2.0)	
60–89	66	7.3 (±2.0)	
30–59	65	8.0 (±2.4)	
< 30	31	7.9 (±2.1)	
MELD score category			0.052
< 20	177	6.7 (±2.2)	
20–29	85	7.4 (±2.3)	
≥ 30	38	7.1 (±2.1)	
Diabetes			0.18
No	208	6.8 (±2.2)	
Yes	79	7.2 (±2.4)	
Hypertension			0.06
No	228	6.8 (±2.3)	
Yes	55	7.4 (±2.4)	
Hepatitis C Virus			0.06
No	150	6.7 (±2.4)	
Yes	139	7.2 (±2.1)	
Mycophenolate mofetil			0.63
No	124	6.8 (±2.4)	
Yes	145	6.9 (±2.2)	
Steroids			0.73
No	21	7.1 (±2.2)	
Yes	259	6.9 (±2.3)	
Calcineurin inhibitor			0.61
Tacrolimus	204	6.9 (±2.3)	
Cyclosporine	20	6.7 (±2.7)	
None	8	7.6 (±2.1)	
Type not known	72	6.9 (±2.1)	

^a^ Units, ml/min/1.73 m^2^

**Fig. 1:**
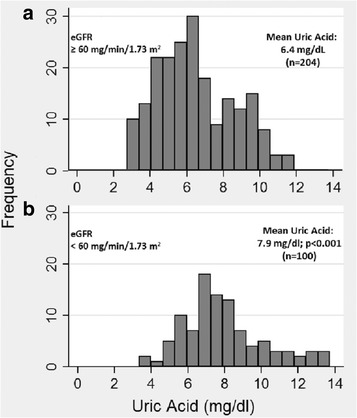
Histograms of uric acid level, according to estimated glomerular filtration rate (eGFR) level, according to eGFR level ≥60 ml/min (Panel **a**) and <60 ml/min (Panel **b**)

The mean (and median) uric acid levels among the 204 patients with an eGFR level ≥60 mg/min/1.73 m^2^ was 6.4 mg/dl (median, 6.1 mg/dl), compared to 7.9 mg/dl (median, 7.6 mg/dl) among the 100 patients with eGFR <60 mg/min/1.73 m^2^ (*p* < 0.001), illustrating a strong association between eGFR at the time of OLT and uric acid level (Fig. 1).

### Uric acid level and survival

During a median of 4.6 years of follow up [minimum, 1 month; maximum, 14.0 years] and a total of 1819 person-years of follow-up from the date of OLT, 89 deaths occurred. Through April, 2010, follow-up mortality data was complete, being assessed through the National Death Index and UNOS registry. The overall crude mortality rate was 48.9 deaths per 1000 person-years [95% confidence interval, 39.7–60.2 deaths/person-years].

**Fig. 2:**
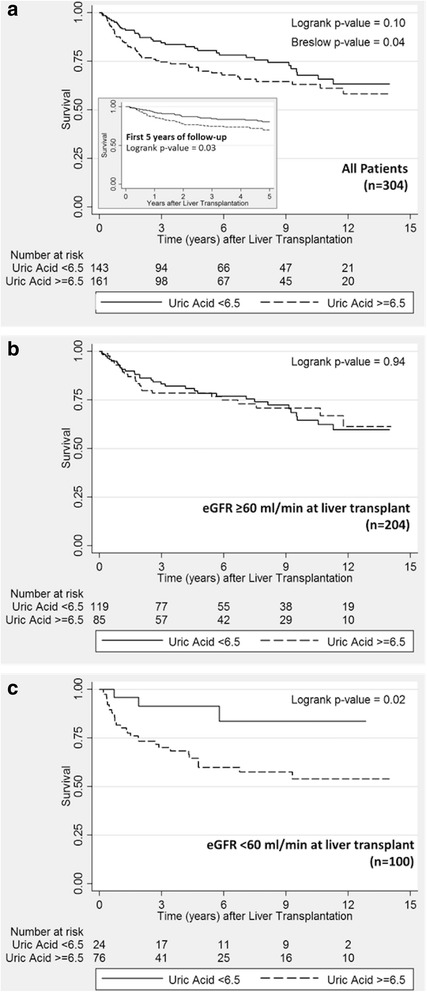
Kaplan-Meier plots of survival after orthotopic liver transplantation, according to mean uric acid level during the first 3 months after liver transplantation and eGFR level at transplant, among all patients with up to 14 years of follow-up (Panel **a**) and up to 5 years of follow-up (Sub-panel *a*); among those with eGFR≥60 ml/min (Panel **b**); and among those with eGFR<60 ml/min (Panel **c**)

The unadjusted mortality rate (Table 3) was higher among those with an elevated uric acid (58.0 deaths per 1000 person-years) than those with a lower uric acid (39.8 deaths per 1000 person-years; *p* = 0.04). A Kaplan-Meier curve of survival according to uric acid level shows early separation (Fig. 2, Panel A), and later narrowing of the two uric acid level curves (Breslow p-value = 0.04). A Kaplan-Meier curve truncated at 5 years of follow-up demonstrates significant separation of the two curves (Log-rank p-value = 0.03). After adjusting for age, gender, and time-varying eGFR level, the overall hazard ratio (HR) of death, while of moderate magnitude (HR = 1.4; confidence interval, C.I. [0.9–2.2]), was not statistically significant (*p* = 0.11). However, when truncated at 5 years of follow-up, the adjusted association becomes significant (HR = 1.7; C.I. [1.0, 2.8]; *p* = 0.045).

**Table 3:** Crude mortality rates and adjusted relative hazard of death after liver transplantation, according to mean uric acid level in the first quarter after transplantation, and stratified by mean eGFR level

	Deaths	Person- years	Crude mortality rate ^a^		Adjusted ^b^ relative hazard of death		
Characteristic				*p*-value	HR	[95% C.I.]	*p*-value
All Patients (*n* = 304)				0.04			
Uric Acid <6.5 mg/dl	36	905	39.8		1.0	Reference	
Uric Acid ≥6.5 mg/dl	53	914	58.0		1.4	[0.9, 2.2]	0.11
Uric Acid (+1 mg/dl increase)					1.07	[0.97, 1.18]	0.16
Stratified analysis							
eGFR ≥ 60 mg/min/1.73 m^2^ (*n* = 204)				0.46			
Uric Acid <6.5 mg/dl	33	750	44.0		1.0	Reference	
Uric Acid ≥6.5 mg/dl	24	530	45.2		1.0 ^c^	[0.6, 1.7]	0.95
Uric Acid (+1 mg/dl increase)					0.9 ^d^	[0.8, 1.1]	0.36
eGFR < 60 mg/min/1.73 m^2^ (*n* = 100)				0.005			
Uric Acid <6.5 mg/dl	3	155	19.3		1.0	Reference	
Uric Acid ≥6.5 mg/dl	29	383	75.6		3.7 ^c^	[1.1, 12.0]	0.03
Uric Acid (+1 mg/dl increase)					1.2 ^d^	[1.1, 1.4]	0.008

^a^ Deaths per 1000 person-years of follow-up

^b^ All models include age, gender, and time-dependent eGFR category

^c^ p-_interaction_ = 0.046

^d^ p-_interaction_ = 0.01

Table 3 also presents a stratified analysis which demonstrates a statistically significant interaction between high uric acid level and low eGFR (p-_interaction_ = 0.046). Among those with eGFR ≥ 60 ml/min/1.73 m^2^, uric acid level does not predict mortality (HR = 1.0; *p* = 0.95). However, among those with an eGFR < 60, a high uric acid level strongly and significantly predicts mortality (HR = 3.7; *p* = 0.03). This finding is also confirmed in the unadjusted stratified analysis of mortality rates (Table 3) and the Kaplan-Meier curves shown in Panels B and C of Fig. 2.

When uric acid was entered into the multivariate model as a continuous variable, it was not associated with mortality among those with a high eGFR. In contrast, a 1 mg/dl higher level of serum uric acid was strongly associated with mortality (HR = 1.2 [1.1, 1.4]; *p* = 0.008) among those with a low eGFR (Table 3). The p-value for interaction in the model with uric acid as a continuous variable was p-_interaction_ = 0.01. When uric acid level was categorized into quartiles (results not shown), none of the estimates was statistically significant, likely owing to insufficient power resulting from the smaller number of deaths in each category, especially among those with lower eGFR and lower uric acid levels.

### Uric acid level and doubling of creatinine

**Fig. 3:**
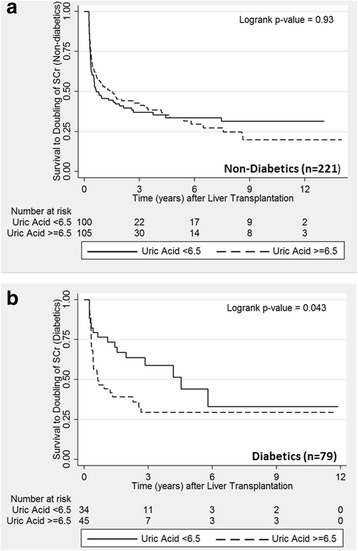
Kaplan-Meier plot of doubling of serum creatinine, according to mean uric acid level during the first 3 months after liver transplantation, and stratified by diabetes status (Non-diabetics, Panel **a**; and Diabetics, Panel **b**)

A total of 180 (60%) participants experienced a doubling of creatinine over 731 person-years and a median of 0.93 years to event or censoring. The overall incidence rate was 278.2 events/1000 person-years. In the entire cohort, elevated uric acid level (either dichotomized or continuous) was not associated with doubling of creatinine (Table 4). However, UA was associated with doubling of creatinine among diabetics (HR = 2.2; *p* = 0.025), but not among non-diabetics (HR = 0.8; *p* = 0.15; p-_interaction_ = 0.061). Similar effect modification by diabetes was seen in the crude analysis of incidence rates (Table 4) and with Kaplan-Meier plots (Fig. 3). There was no interaction according to baseline eGFR level (p-_interaction_ = 0.38).

**Table 4:** Crude rates and adjusted relative hazards of doubling of creatinine and USRDS-documented progression to end-stage renal disease (ESRD) after liver transplantation, according to mean uric acid level in the first quarter after transplantation

	Events	Person- years	Crude rate ^a^		Adjusted ^b^ Relative Hazard		
Characteristic				*p*-value	HR	[95% C.I.]	*p*-value
Outcome: doubling of creatinine							
All Patients (*n* = 300)				0.24			
Uric Acid <6.5 mg/dl	81	361	224.3		1.0	Reference	
Uric Acid ≥6.5 mg/dl	99	370	267.3		0.9	[0.7, 1.3]	0.70
Uric Acid (+1 mg/dl increase)					0.96	[0.90, 1.03]	0.30
Analysis stratified by diabetes							
Non-diabetics(*n* = 221)				0.86			
Uric Acid <6.5 mg/dl	63	252	249.5		1.0	Reference	
Uric Acid ≥6.5 mg/dl	69	268	257.2		0.8 ^c^	[0.5, 1.1]	0.15
Uric Acid (+1 mg/dl increase)					0.92 ^d^	[0.84, 0.99]	0.04
Diabetics(*n* = 79)				0.014			
Uric Acid <6.5 mg/dl	16	100	160.7		1.0	Reference	
Uric Acid ≥6.5 mg/dl	29	85	341.1		2.2 ^c^	[1.1, 4.3]	0.025
Uric Acid (+1 mg/dl increase)					1.1 ^d^	[1.00, 1.31]	0.049
Outcome: Progression to ESRD							
All Patients (*n* = 300)				0.31			
Uric Acid <6.5 mg/dl	16	844	18.9		1.0	Reference	
Uric Acid ≥6.5 mg/dl	19	845	22.5		0.9	[0.4, 1.7]	0.70

^a^ Events (doubling of Scr or progression to ESRD, respectively) per 1000 person-years of follow-up

^b^ All models include age, gender, and time-dependent eGFR category

^c^ p-_interaction_ = 0.061

^d^ p-_interaction_ = 0.042. No interactions with eGFR or diabetes were seen in the association between UA and ESRD, and no interactions with eGFR were seen for doubling of creatinine

### Uric acid level and ESRD incidence

During a median of 4.4 years of follow up [minimum, 1 month; maximum, 14.0 years] and a total of 1689 person-years of follow-up from the date of OLT through April, 2010, 35 (11.7%) of the 300 patients under follow-up were enrolled into the Medicare dialysis program. The overall ESRD incidence rate was 20.7 events per 1000 person-years [95% confidence interval, 14.8–28.9 events/1000 person-years].

The unadjusted ESRD incidence rate (Table 4) was similar among those with elevated uric acid compared to those with lower uric acid. All adjusted associations were null, whether uric acid was modeled as a dichotomous, continuous variable or a quartile variable; and no interactions by eGFR or diabetes were seen (Additional file 1: Table S1). A Kaplan-Meier curve of survival according to uric acid level confirmed no difference in ESRD occurrence according to uric acid level (*p* = 0.65; figure not shown).

Analyses stratified a priori by eGFR level and separately stratified a priori by diabetes status demonstrated no subgroups with a significant association with uric acid level. No significant interactions with eGFR level or diabetes were present. Similar null findings were found when uric acid was entered into the analogous multivariate models as a continuous variable.

Other predictors significantly associated with ESRD after OLT included diabetes, MELD score category, eGFR category at time of OLT, and presence of hepatorenal syndrome at time of OLT (Table 5). The presence of hepatitis C was associated with ESRD (HR = 1.9), but the association did not reach statistical significance. End-stage renal disease was not significantly associated with age, sex, race, cause of liver failure, hypertension, hepatocellular carcinoma, mycophenolate mofetil use, steroid use, or type of CNI used.

**Table 5:** Predictors of USRDS-documented end-stage renal disease (ESRD) after liver transplantation

	Adjusted ^b^ Relative Hazard of ESRD		
Characteristic	HR	[95% C.I.]	*p*-value
Diabetes			
No	1.0	Reference	
Yes	2.6	[1.3, 5.2]	0.005
MELD score category			
< 20	1.0	Reference	
20–29	2.2	[1.0, 4.7]	0.04
≥ 30	2.6	[1.0, 6.4]	0.04
	*p-trend = 0.02*		
MELD Era			
Pre-MELD era (<2/02)	1.0	Reference	
MELD era (≥2/02)	2.0	[0.8, 5.1]	0.13
eGFR category at OLT			
≥ 90	1.0	Reference	
60–89	2.6	[0.9, 7.0]	0.07
30–59	2.6	[1.0, 7.0]	0.06
< 30	4.9	[1.6, 14.6]	0.005
	*p-trend = 0.006*		
Hepatorenal syndrome			
No	1.0	Reference	
Yes	3.8	[1.4, 10.3]	0.01
Hepatitis C virus infection			
No	1.0	Reference	
Yes	1.9	[1.0, 3.9]	0.065
Calcineurin inhibitor use^b^			
Tacrolimus	1.0	Reference	
Cyclosporine	1.0	[0.4, 3.1]	0.94
Type not known	0.7	[0.3, 1.6]	0.40

^a^ All models include age, gender, diabetes, and time-dependent eGFR category. Note: ESRD was not significantly associated in the adjusted models with age, sex, race, cause of liver failure, hypertension, hepatocellular carcinoma, mycophenolate mofetil use, or steroid use

^b^ The “None” category for CNI use had too few observations for inclusion in the model

## Discussion

This study examines the association between hyperuricemia and mortality, doubling of creatinine, and ESRD incidence after OLT. We found that over a median follow up period of 4.6 years, a serum uric acid level greater than or equal to 6.5 mg/dL was independently associated with an almost a four-fold increase in mortality, but only among those with GFR < 60 mL/min/1.73 m^2^ (HR = 3.7, *p* = 0.03), and not those with GFR ≥60 mL/min/1.73 m^2^. The study also found that elevated UA levels are associated with doubling of creatinine among diabetics (HR = 2.2, *p* = 0.025), but not among diabetics. Although these findings do not infer causality, they lay the groundwork for future intervention studies among OLT patients with both hyperuricemia and diminished renal function to establish whether mortality or progression of renal disease in diabetics can be reduced with treatment of hyperuricemia. Conversely, no association was found between hyperuricemia and the development of ESRD, perhaps owing to the small number of ESRD events with resultant limited power.

Although the association between increased mortality and hyperuricemia has been reported in other transplant populations such as renal [14] and heart transplant recipients, [19] this is the first study to investigate the effect of hyperuricemia on mortality among orthotopic liver transplant recipients. Dahle et al. reported that in renal transplant recipients, a J-shaped association exists between serum uric acid level and cardiovascular and all-cause mortality [14]. Our study did not identify such a J-shaped association between uric acid level and mortality, although it should be noted that the association of the lowest uric acid levels with mortality reported by Dahle was statistically non-significant (RH = 1.31; *p* = 0.18). Similar to our study, however, Arora, et al.*,* found increased mortality among heart transplant patients with elevated serum uric acid levels at 1 year, [19] although they did not stratify the association according to eGFR level. The cause of the observed effect modification by eGFR level is not known, although one may hypothesize that patients with low eGFR may be more prone to hyperuricemia-induced acceleration of endothelial and vascular injury. Importantly, since uric acid level increases with decreases in renal function, it is difficult to disentangle the association of uric acid with outcomes associated with decreased renal function. While our retrospective study adjusted for time-dependent eGFR, such adjustment may be incomplete owing to gaps in renal function measurements in clinical practice. A prospective cohort study which measures renal function regularly on all patients should be performed in the future to provide the strongest evidence of the role of uric acid in these outcomes.

The association between hyperuricemia and mortality has been postulated to be related to increased cardiovascular events in those with high uric acid levels [20]. Studies in non-transplant populations, however, have not demonstrated such an association. The Framingham Heart Study group concluded that uric acid is merely a marker of increased risk for cardiovascular disease due to its association with hypertension, hyperlipidemia and impaired glucose metabolism [21]. In our study, information regarding cause-specific mortality was not available and hence we cannot determine if the increased mortality associated with hyperuricemia in the low-eGFR group was related to cardiovascular events.

The present study found that OLT recipients with GFR < 60 mL/min/1.73 m^2^ had significantly higher mean serum uric acid in the post-transplant period compared to recipients with GFR ≥ 60 mL/min/1.73 m^2^. This inverse association emphasizes the central role of the kidney in urate clearance and the dose–response association between urate levels and renal function in the OLT population, which renders the disentanglement of the two effects on renal and other outcomes challenging.

Our study demonstrated no overall association between UA level and the incidence of ESRD or doubling of creatinine. However, we did find that UA level predicts doubling of creatinine among diabetics. Historically, uric acid has been identified only as a marker of renal damage. However, some observational studies have raised the possibility that the relationship between renal function and uric acid may be more complex [22, 23]. Uric acid was hypothesized to play a role in causing renal dysfunction by induction of afferent arteriolopathy, inflammation, and activation of the renin-angiotensin system [24]. Supporting this notion is the observation that renal function improves among liver transplant patients with gout and hyperuricemia after treatment with allopurinol, a urate-lowering medication [3]. While this study cannot assess the mechanism for the observed interaction with diabetes, it does support the hypothesis that UA may increase the deleterious effect of diabetes on the kidney.

The incidence of post-OLT ESRD has been previously estimated at 12.8 and 14.5 per 1000 patient-years in the pre- and post-MELD era, respectively [25]. The overall incidence of ESRD in our cohort was higher, at 20.7 per 1000 years, though only 35 patients progressed to ESRD.

Some other studies have suggested various factors potentially contribute to the progression of renal disease and ESRD development in patients after OLT, including hyperuricemia [26]. Several studies in the general population have demonstrated an association between hyperuricemia and ESRD [9, 27] but others did not find this association, especially in advanced stages of CKD (stage III-V) [28]. The small number of ESRD events in our study and the resultant low statistical power may partially explain the lack of association between hyperuricemia and ESRD incidence in our cohort, although the associations between uric acid (as a dichotomous, quartile, and continuous variable) and ESRD incidence were all very close to the null. The factors associated with increased ESRD incidence in our study included lower eGFR, diabetes, higher MELD score, and the presence of hepatorenal syndrome, all of which are pathophysiologically plausible.

Calcineurin inhibitors, particularly cyclosporine, have been cited as a factor that could lead to hyperuricemia after organ transplantation [29]. Cyclosporine can cause hyperuricemia by increasing net tubular urate reabsorption [30] or decreasing the glomerular filtration of uric acid [31]. However, our study did not find associations between CNI sub-type and either uric acid level after OLT or ESRD incidence, perhaps because the frequency of cyclosporine use was less than 10% in this cohort and we were not able to take the effects of CNI use on UA fully into account. Use of allopurinol to lower uric acid levels in this cohort was also very low, so use of urate-lowering medications is unlikely to have confounded these results.

A number of plasma membrane transporter proteins participate in uric acid handling. Among the various transporter proteins that play the most important role in uric acid reabsorption are URAT1 protein which is only expressed in human kidney on the brush border membrane of the proximal tubule and GLUT9 which is expressed in the basolateral membrane of the proximal tubule but also the basolateral membrane of hepatocytes [4]. Studies on diverse patients’ population have identified mutations or allelic variants in genes encoding for specific urate renal transporters that are associated with hyperuricemia as a result of urate under-excretion [32–34]. In our study, genetic testing for these conditions was not performed. However, this may be worthwhile in future studies, since these genes could be potential targets for treatment.

The present study is limited by lack of cause-specific mortality during the follow-up period. Nevertheless, this study is important, as it is the only study to examine the impact of hyperuricemia on total mortality, doubling of creatinine and incidence of ESRD among liver transplant patients. Second, uric acid levels were only reliably available for 3 months post OLT and we could not exclude the possibility of residual confounding. Nonetheless, the results of this study could provide a rationale for interventional studies assessing the role of early treatment of hyperuricemia in liver transplant recipients and its effect on mortality, particularly among those with renal dysfunction; and its effect on progression of renal disease among those with diabetes. Recently, treatment of asymptomatic hyperuricemia post kidney transplantation was associated with a substantial benefit in patient and graft survival [15]. Another limitation is that GFR estimating equations do not perform very well in patients with liver disease, thus resulting in the potential of some misclassification of renal status above and below the eGFR cut-point value of 60 mg/min/1.73 m^2^. Also, the 14-year follow-up includes a period during which transplant practices have changed, the effects on outcomes of which were likely not fully captured in this retrospective study. We also did not have information on type of CNI used in 23% of the cohort, which could bias the findings related to CNI use in an unknown direction. Lastly, the limitation of CNI subtype to 2003 and later may result in an underestimation of the use of cyclosporine, since some patients on cyclosporine before 2003 may have switched to tacrolimus. If this occurred to a significant degree, this would bias the association of CNI subtype with ESRD incidence towards the null.

## Conclusions

In conclusion, mean serum uric acid levels are elevated after orthotopic liver transplantation, and are significantly associated with eGFR category in the OLT population. Hyperuricemia is independently associated with mortality, particularly among liver transplant patients with GFR less than 60 mL/min/1.73 m^2^, and associated with doubling of creatinine among diabetics, but is not associated with ESRD incidence.

## Additional file

Additional file 1: Table S1. (Extension of Table 4). Crude rates of progression and adjusted relative hazards of USRDS-documented end-stage renal disease (ESRD) after liver transplantation, according to mean uric acid level in the first quarter after transplantation, and stratified by mean eGFR level and diabetes. (DOCX 19 kb)

## Abbreviations

ANOVA: Analysis of variance
CKD: Chronic kidney disease
CKD-EPI: Chronic Kidney Disease Epidemiology Collaboration
CNI: Calcineurin inhibitor
eGFR: Estimated glomerular filtration rate
ESRD: End-stage renal disease
HR: Hazard ratio
INR: International normalized ratio
MELD: Model for end-stage liver disease
OLT: Orthotopic liver transplantation
UA: Uric acid
UNOS: United Network for Organ Sharing
USRDS: United States Renal Data System
