# Supplementary material for: Hyperuricemia after orthotopic liver transplantation: divergent associations with progression of renal disease, incident end-stage renal disease, and mortality
Source: BMC Nephrol. 2017 Mar 27;18:103. doi: 10.1186/s12882-017-0518-5 (PMC5369182; doi:10.1186/s12882-017-0518-5)
Supplement: Additional file 1: Table S1. — (Extension of Table 4). Crude rates of progression and adjusted relative hazards of USRDS-documented end-stage renal disease (ESRD) after liver transplantation, according to mean uric acid level in the first quarter after transplantation, and stratified by mean eGFR level and diabetes. (DOCX 19 kb) [file 12882_2017_518_MOESM1_ESM.docx]

**Table S1 (Extension of Table 4).** Crude rates of progression and adjusted relative hazards of USRDS-documented end-stage renal disease (ESRD) after liver transplantation, according to mean uric acid level in the first quarter after transplantation, and stratified by mean eGFR level.

|  | **Number Progressing to ESRD** | **Person- Years** | **ESRD**  **Incidence**  **Rate ^a^** |  |  | **Adjusted** **^b^ Relative  Hazard of ESRD** | |  |
| --- | --- | --- | --- | --- | --- | --- | --- | --- |
| **Characteristic** |  |  |  | **p-value** |  | HR | [95% C.I.] | p-value |
|  |  |  |  |  |  |  |  |  |
| **All Patients** (n=300) |  |  |  | 0.31 |  |  |  |  |
| Uric Acid <6.5 mg/dl | 16 | 844 | 18.9 |  |  | 1.0 | Reference |  |
| Uric Acid ≥6.5 mg/dl | 19 | 845 | 22.5 |  |  | 0.9 | [0.4, 1.7] | 0.70 |
|  |  |  |  |  |  |  |  |  |
|  |  |  |  |  |  |  |  |  |
| Uric Acid <5.5 mg/dl | 7 | 471 | 14.8 | Ref. |  | 1.0 | Reference |  |
| Uric Acid 5.5–6.4 mg/dl | 9 | 372 | 24.1 | 0.35 |  | 1.2 | [0.4, 3.4] | 0.70 |
| Uric Acid 6.5–8.4 mg/dl | 9 | 471 | 19.1 | 0.63 |  | 0.7 | [0.2, 2.1] | 0.54 |
| Uric Acid ≥8.5 mg/dl | 10 | 374 | 26.7 | 0.24 |  | 1.4 | [0.5, 3.8] | 0.52 |
|  |  |  |  |  |  |  |  |  |
| Uric Acid (+1 mg/dl increase) |  |  |  |  |  | 1.1 | [0.9, 1.3] | 0.20 |
|  |  |  |  |  |  |  |  |  |
| **Stratified by eGFR category** |  |  |  |  |  |  |  |  |
| eGFR ≥ 60 mg/min/1.73m^2^ (n=202) |  |  |  | 0.60 |  |  |  |  |
| Uric Acid <6.5 mg/dl | 9 | 711 | 12.7 |  |  | 1.0 | Reference |  |
| Uric Acid ≥6.5 mg/dl | 8 | 486 | 16.4 |  |  | 1.1 ^c^ | [0.4, 2.9] | 0.90 |
|  |  |  |  |  |  |  |  |  |
| Uric Acid (+1 mg/dl increase) |  |  |  |  |  | 1.1 ^d^ | [0.9, 1.4] | 0.30 |
|  |  |  |  |  |  |  |  |  |
| eGFR < 60 mg/min/1.73m^2^ (n=98) |  |  |  | 0.28 |  |  |  |  |
| Uric Acid <6.5 mg/dl | 7 | 133 | 52.6 |  |  | 1.0 | Reference |  |
| Uric Acid ≥6.5 mg/dl | 11 | 358 | 30.7 |  |  | 0.4 ^c^ | [0.1, 1.0] | 0.06 |
|  |  |  |  |  |  |  |  |  |
| Uric Acid (+1 mg/dl increase) |  |  |  |  |  | 0.9 ^d^ | [0.7, 1.2] | 0.59 |
|  |  |  |  |  |  |  |  |  |
| **Stratified by Diabetes** |  |  |  |  |  |  |  |  |
| No Diabetes (n=205) |  |  |  | 0.38 |  |  |  |  |
| Uric Acid <6.5 mg/dl | 8 | 638 | 12.5 |  |  | 1.0 | Reference |  |
| Uric Acid ≥6.5 mg/dl | 11 | 579 | 19.0 |  |  | 1.5 ^e^ | [0.6, 3.8] | 0.43 |
|  |  |  |  |  |  |  |  |  |
| Uric Acid (+1 mg/dl increase) |  |  |  |  |  | 1.2 ^f^ | [0.9, 1.4] | 0.16 |
|  |  |  |  |  |  |  |  |  |
| Diabetes (n=79) |  |  |  | 0.39 |  |  |  |  |
| Uric Acid <6.5 mg/dl | 8 | 149 | 53.7 |  |  | 1.0 | Reference |  |
| Uric Acid ≥6.5 mg/dl | 8 | 231 | 34.5 |  |  | 0.4 ^e^ | [0.1, 1.2] | 0.10 |
|  |  |  |  |  |  |  |  |  |
| Uric Acid (+1 mg/dl increase) |  |  |  |  |  | 1.0 ^f^ | [0.8, 1.3] | 0.73 |
|  |  |  |  |  |  |  |  |  |
|  |  |  |  |  |  |  |  |  |

^a^ Deaths per 1000 person-years of follow-up. ^b^ All models include age, gender, diabetes, and time-dependent eGFR category.
^c^ p_interaction_ = 0.14. ^d^ p_interaction_ = 0.34; ^e^ p_interaction_ = 0.20. ^f^ p_interaction_ = 0.57.
